# EHMTI-0131. Clinical features of cluster headache in women

**DOI:** 10.1186/1129-2377-15-S1-C3

**Published:** 2014-09-18

**Authors:** C Tassorelli, Y Falzone, R De Icco, MG Cuzzoni, G Nappi, G Sances

**Affiliations:** 1 Headache Science Center C. Mondino National Neurological Institute, Dept of Brain and Behaviour University of Pavia, Pavia, Italy; 2 Headache Science Center, C. Mondino National Neurological Institute, Pavia, Italy

## Introduction

Cluster Headache (CH) mostly affects men but a substantial percentage of women also suffer this headache disorder. Little is known about possible gender-related differences in the characteristics of attacks from studies where CH diagnosis was validated.

## Aim

To evaluate retrospectively the differences in demographics, headache characteristics, concomitant diseases and treatment response of 198 CH patients diagnosed and followed at the Pavia Headache Centre.

## Results

Data from 134 males and 64 females were collected. The mean age at CH onset was lower in women than in men (24.8±10.8y vs 28.03±10.2y, p<0.43). Episodic form of the disease was diagnosed in 91% of subjects, without gender difference. No differences were detected as regards the annual mean number of CH periods, their mean duration and the average daily frequency of attacks during the active phase. Untreated attacks were shorter in men (90 minutes vs 107 minutes, p<0.02). A family history of migraine was present in 71.4% of women and 59.1% of men (p=0.06). Nausea, vomiting, photo and osmophobia were reported more frequently by women than men, while local autonomic symptoms were almost equally distributed between sexes. No difference was found in treatment response between genders. Female CH sufferers presented more frequently thyroid disorders and psychiatric illness than men. On the contrary, snoring in sleep occurred statistically more frequently in men.

## Conclusions

This retrospective survey shows some specific features for CH in women: earlier onset of disease, more frequent association with 'migrainous' symptoms during the attacks and a longer duration of untreated attacks.

